# Electrochemical Properties of Nitrate-Selective Electrodes: The Dependence of Resistance on the Solution Concentration

**DOI:** 10.3390/s18072062

**Published:** 2018-06-28

**Authors:** Arina Ivanova, Konstantin Mikhelson

**Affiliations:** Chemistry Institute, St. Petersburg State University, 199034 Saint Petersburg, Russia; rosengartenchen@gmail.com

**Keywords:** nitrate-selective electrode, potentiometric response, bulk and interfacial resistance, capacitance, charged species concentrations, diffusion coefficients, chronopotentiometry, electrochemical impedance

## Abstract

The electrochemical properties of ion-exchanger-based solvent polymeric ion-selective electrodes (ISEs)—bulk and interfacial resistance, capacitance, and polarization under a galvanostatic current step—are studied, with a nitrate ISE based on tetradecylammonium nitrate (TDANO_3_) as a model system. The study is performed by chronopotentiometric and impedance measurements, and focuses on the dependence of the aforementioned properties on the concentration of NO_3_^−^ anions in solution. The impacts from the bulk and the interfacial charge transfer to the overall membrane resistance are revealed. It is shown that the bulk resistance of the membranes decreases over an increase of NO_3_^−^ concentration within the range of a Nernstian potentiometric response of the ISE. This fact, also reported earlier for K^+^- and Ca^2+^-selective ISEs, is not in line with current views of the mechanism of the ISE response, or of the role of ion exchange in particular. The origin of this effect is unclear. Estimates are made for the concentration of ionized species (NO_3_^−^ and TDA^+^) and, respectively, for the TDANO_3_ association constant, as well as for the species diffusion coefficients in the membrane.

## 1. Introduction

Ion-selective electrodes (ISEs) with solvent-polymeric membranes are routinely used as sensors of ion activity in a broad range of applications, primarily in clinical chemistry, environmental monitoring, and industry. The classical theory of the ISE response focuses primarily on ion-exchange processes at the interface between the membrane and aqueous solution. It also takes into consideration a co-extraction of the aqueous electrolyte from the sample or calibration solution, which governs the upper limit of the ISE response [1,2,3,4]. More recently, it was shown that the lower limit of the ISE response is due to trans-membrane fluxes of electrolyte co-extracted from the internal solution [5,6,7], or from the internal reference system in the case of solid-contact ISEs [8]. Among numerous approaches aimed at the improvement of the lower detection limit, galvanostatic polarization appears the most flexible [9,10,11,12,13]. Recently, we demonstrated that galvanostatic polarization also allows for the improvements for the upper detection limit of ISEs [14].

Advanced models of ISEs cover both lower and upper limits of their response and allow for the description of the ISE potential for fully dissociated membranes (no ion pairs) over real time and space [15,16,17,18]. A more idealized model is also capable of describing the impact of co-extraction to the limits and the slope of the ISE response within the linear range, although only for two monovalent ions at a steady state [19]. The model has been successfully verified using a K^+^-selective valinomycin-based ISE. A significant decrease of the membrane bulk resistance close to the upper limit of the ISE response was registered and explained as a result of co-extraction of the electrolyte from solution.

It is widely recognized that within the Nernstian response range co-extraction plays a minor role or is negligible. Also, within this range only the boundary potential at the membrane/sample interface varies, whereas other contributions to the overall membrane potential and to the EMF of the galvanic cell are essentially constant. In turn, the boundary potential follows the equation

(1) $$\varphi_{b}=\frac{\mu_{I}{}^{0,aq}-\mu_{I}{}^{0,mem}}{F}+\frac{RT}{z_{I}F}\ln\frac{a_{I}{}^{aq}}{a_{I}{}^{mem}}$$

Here $\varphi_{b}$ is the boundary potential; $\mu_{I}{}^{0,aq},\;\mu_{I}{}^{mem}$ are the standard chemical potential values; $a_{I}{}^{aq},\;a_{I}{}^{mem}$ are the activities of $I^{Z_{I}}$ analyte ion in the aqueous and membrane phases, respectively; $z_{I}$ is the ion charge; and *F*, *R*, and *T* are Faraday constant, gas constant, and temperature, respectively. Equation (1) transforms into

(2) $$\varphi_{b}=\varphi_{b}{}^{0}+\frac{RT}{z_{I}F}\ln a_{I}{}^{aq}$$

with a constant $\varphi_{b}{}^{0}$ only if $a_{I}{}^{mem}$ is constant. Respectively, the composition of the membrane within the Nernstian response range must be constant. Essentially, this means that the membrane bulk resistance must be also constant, so that a Nernstian-responding sensor should not show a conductometric response, and vice versa.

However, it was reported that K^+^ ISEs based on valinomycin may show both a potentiometric and a conductometric response [20]. A recent systematic study of the electrochemical properties of Ca^2+^ ISEs based on ETH 1001 also showed a non-constancy of the membrane bulk resistance within the Nernstian response range [21]. These findings may put into question the established views on the mechanism of the ISE response, which is of an academic, and also a practical interest. To the best of our knowledge, anion-selective ISEs have not been studied in this respect. Therefore, in this paper we study the electrochemical properties of anion-selective ISEs based on simple ion exchangers (Hofmeister ISEs) using NO_3_^−^-ISE based on tetradecylammonium nitrate as a model system.

## 2. Materials and Methods

The membrane consisted of 32.6% poly(vinylchloride) (PVC) *S-66* (high molecular weight) made by Ohtalen (St.Petersburg, Russia), 65.2% solvent-plasticizer bis(2-ethylhexyl)phthalate (DOP) obtained from Reaktiv (Moscow, Russia), and 2.2% (0.05 M) anion exchanger tetradecylammonium bromide (TDABr) from Analiz-X (Minsk, Belarus). Tetrahydrofuran (THF) and cyclohexanone (CH) were from Vecton (Russia), and distilled before use. KNO_3_ and KCl were obtained from Reaktiv (Moscow, Russia). All aqueous solutions were prepared with deionized (DI) water with resistivity 18.2 MΩ∙cm (Milli-Q Reference, Millipore, France). The membrane cocktail was prepared by dissolving appropriate amounts of PVC, DOP, and TDABr in THF. The “dry” content in the cocktail was 18%. To obtain the membrane, the cocktail was stirred for 30 min using roller-mixer Movil Rod Selecta (Barcelona, Spain) and then cast on a glass Petri dish with diameter of 70 mm. The dish was closed with filter paper to slow down the evaporation of THF. The complete evaporation of THF took 1 day, and in this way a master membrane with a thickness of 0.7 mm was obtained.

The electrodes were prepared by cutting disks with a diameter of 12 mm from the master membrane and gluing them to PVC bodies with an outer diameter of 12 mm and an inner diameter of 10 mm. A solution of PVC in CH was used as the glue. The internal reference electrode was chlorinated silver wire in a polypropylene body. The electrode design is shown in Figure S1 in the Supplementary Materials.

Initially, the ISEs were filled with 0.1 M KNO_3_ and conditioned in this solution for two weeks. Every other day during this time the solution was replaced with a fresh portion. This was done to replace Br^−^ in the membrane phase with NO_3_^−^ anions. After that, the ISEs were thoroughly rinsed with DI water, filled with mixed solution containing 0.01 M KNO_3_ and 0.001 M KCl, and conditioned in the same solution for one week. The chloride background was needed for the reliable work of the internal Ag/AgCl electrode.

Zero current potentiometric measurements were performed with Ecotest-120 eight-channel potentiometric station Econics (Moscow, Russia). The reference electrode was a single junction Ag/AgCl electrode in 3.5 M KCl, with a salt bridge and a limited leak of KCl. Calibration in KNO_3_ solutions was performed from 0.1 M down to 10^−8^ M KNO_3_ using automatic diluter 700 Dosino controlled by the 711 Liquino Controller (Metrohm, Buchs, Switzerland). Chronopotentiometric curves and electrochemical impedance spectra were recorded with a Potentiostat-Galvanostat Autolab 302N with a frequency response analyzer module FRA 2 (Metrohm, Buchs, Switzerland). The chronopotentiometric and impedance measurements were carried out using symmetric cells with equal compositions of the internal and the external solutions, using an Ag/AgCl electrode (chlorinated silver wire) immersed in these solutions. The counter electrode was glassy carbon rod.

In chronopotentiometric measurements, the electrode potential was initially registered for 10 s without current. Then the current value was abruptly changed from zero to 10^−8^ A (the respective current density was 2.88 × 10^−8^ A/cm^2^) and the potential was registered for 60 s. After that the current was turned off, and the potential was registered for another 60 s. The time resolution in chronopotentiometric measurements was always 0.2 s. The impedance measurements were made in potentiostatic mode with an excitation magnitude of ±5 mV around the open circuit potential, over the frequency range from 100 kHz to 0.01 Hz.

All measurements were carried out in a plastic beaker with a volume of 50 mL, at room temperature (22 ± 1 °C). Three replicate electrodes (ISE 1, ISE 2, and ISE 3) were used in this work. Activities of the NO_3_^−^ anion in solution were calculated according to the second approximation of the Dubye–Hückel theory:

(3) $$\log\gamma_{NO3}=-\frac{0.512\sqrt{J}}{1+a_{Kjel}\cdot 0.328\sqrt{J}}$$

Here $\gamma_{I}$ is the ion activity coefficient, *J* is the solution ionic strength, and $a_{Kiel}$ is the Kielland parameter (equal to 3 for NO_3_^−^) [22].

## 3. Results

### 3.1. Potentiometric Measurements

NO_3_^−^-ISEs with membranes based on quaternary ammonium salts have been known since the 1970s. These ISEs have been thoroughly characterized with regard to their potentiometric response and selectivity, and widely used in practical applications [1,23,24]. Therefore, our potentiometric studies were limited to check the linear range of the ISEs response in KNO_3_ solutions and the selectivity to NO_3_^−^ over Cl^−^. One should expect that the use of nitrate salts with different cations results in different values for the upper limit of the linear response of the ISE [1,2,3,4]. This limit should be lower in the case of CsNO_3_ and higher in the case of LiNO_3_. However, our goal was to see whether the bulk resistance of the membrane depends or does not depend on the concentration of the solution within the linear response range. This is why the studies have been focused on KNO_3_ solutions.

**Figure 1:**
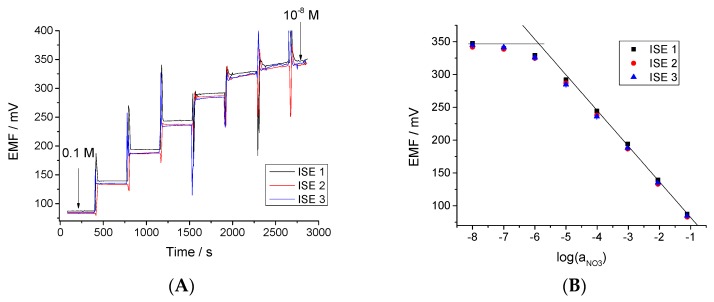
(**A**) Ion-selective electrode (ISE) response curves over the sequential 10-fold dilution of 0.1 M KNO_3_ with deionized (DI) water; (**B**) the respective calibration curve. The straight lines in (**B**) refer to ISE 1.

The response curve obtained by sequential 10-fold dilution of 0.1 M KNO_3_ with DI water down to 10^−8^ M is shown in Figure 1A. In each solution, the EMF was recorded for 300 s, and the average signal for the last 100 s was used to plot the calibration curve shown in Figure 1B. The ISEs showed a linear response to NO_3_^−^ from 0.1 to 10^−5^ M NO_3_^−^; the lower detection limit was log(a_NO3_) = −5.9, and a further decrease of the NO_3_^−^ concentration results in a saturation of the response. The slopes within the linear range were −53.7 ± 0.4 mV/$\log(a_{NO3})$. These slightly sub-Nernstian slopes are common for NO_3_^−^-ISEs based on quaternary ammonium salts [4,25]. The selectivity of NO_3_^−^ over Cl^−^ (separate solutions method) showed that log*K*_NO3/Cl_ ≈ −1.8.

### 3.2. Chronopotentiometric and Impedance Measurements

Chronopotentiometric and impedance techniques have been used for the measurements of the resistances and other electrochemical parameters of the ISE membranes. Membranes contact two aqueous solutions (internal and external), and the respective interfaces contribute to the measured signals. Therefore, a correct interpretation of a chronopotentiometric curve or an impedance spectrum can be performed only for a symmetric cell: the internal and the external solutions must be the same, as well as the respective reference electrodes [19,21,26]. The internal and external reference electrodes were chlorinated silver wires, and therefore the nitrate solutions used in this part of the study always contained a 0.001 M KCl background, to ensure the stability of the Ag/AgCl electrodes. The selectivity of the ISEs allowed for neglecting the presence of 0.001 M Cl^−^ at nitrate concentration down 10^−4^ M. Therefore the chronopotentiometric and impedance studies covered the NO_3_^−^ concentrations from 10^−1^ to 10^−4^ M.

#### 3.2.1. Chronopotentiometric Measurements

**Figure 2:**
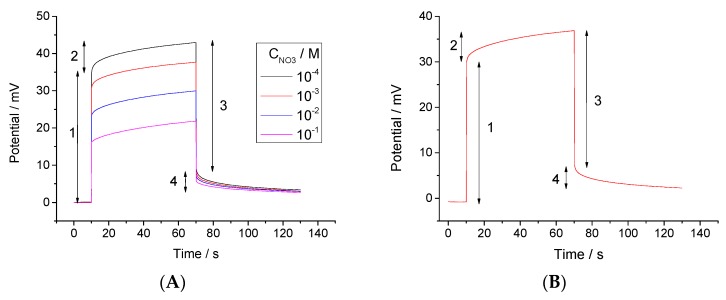
(**A**) Chronopotentiometric curves obtained with ISE 1 after five days soaking in the respective solutions; 1: positive Ohmic drop, 2: polarization, 3: negative Ohmic drop, 4: relaxation—all for 10^−4^ KNO_3_; (**B**) The dependence of the Ohmic drop value in 0.001 M KNO_3_ on the soaking time.

Chronopotentiometric curves obtained with ISE 1 are shown in Figure 2A. Before the current was turned on, the potential was almost zero, due to the symmetry of the cell. When the polarizing current was turned on a positive Ohmic potential drop was observed, followed by a polarization curve. A negative Ohmic drop followed, with a relaxation curve registered after the current was turned off. The magnitudes of the positive and the negative Ohmic drops were the same within ±2–3%. In studies with Ca^2+^-ISEs [21], the resistance calculated from these drops were consistent with the high-frequency resistances obtained in the impedance measurements. However, NO_3_^−^-ISEs showed a more complicated behavior (see below). Also, unlike the case of Ca^2+^-ISEs, obtaining reproducible data in chronopotentiometric (and also in impedance) measurements when one solution is replaced with another required several days of soaking. Example of how the magnitude of the positive Ohmic drop when 10^−4^ M KNO_3_ was replaced with 10^−3^ M solution is shown in Figure 2B. The data shown in Figure 2A and all further data refer to measurements performed after five days of soaking the ISEs in the respective solutions.

The values of R_chrono_—the resistance obtained from the Ohmic drop values—are presented in Table S1.

The polarization and relaxation parts of the chronopotentiometric curves recorded with all ISEs in all solutions were successfully fitted to the following equation:

(4) $$\eta =iR_{\mathrm{expon}}\left(1-\exp\left(-t/\tau \right)\right)+iN\sqrt{t}$$

**Figure 3:**
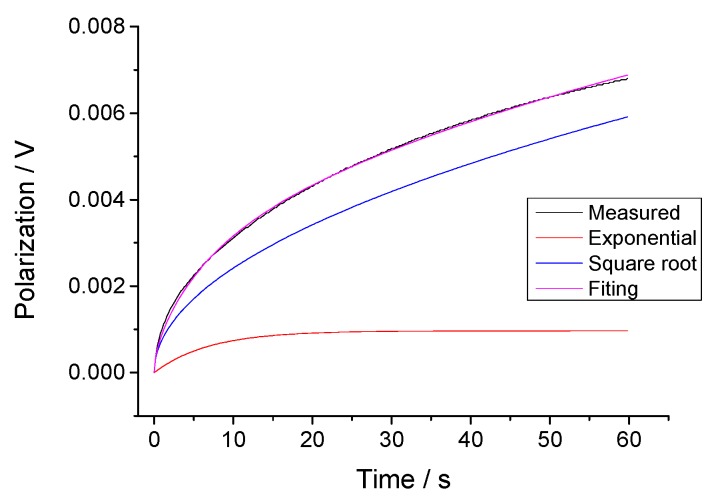
Fitting of the polarization curve of ISE 2 in 0.01 M KNO_3_.

Here *η* is the polarization (or relaxation); *i* is the polarizing current density; *R*_expon_ is the resistance, which refers to the decaying exponent with *τ* as the characteristic time; *t* is time from the moment when current was turned on or off; and *N* is a coefficient related to the transportation impact to polarization or relaxation. Fitting was performed with OriginLab Origin 9 software, and the measured data were fitted to a function defined as $y=A\left(1-\exp\left(-x/\tau \right)\right)+B\sqrt{x}$, where y was the polarization or relaxation value (V) and *x* was the time value (s). In this way we obtained $R_{\mathrm{expon}}=A/i$. An example of the experimental and fitted curves is shown in Figure 3.

In this way we obtained the values of the exponential part of the resistance, $R_{\mathrm{expon}}{}^{\mathrm{polar}}$ and $R_{\mathrm{expon}}{}^{\mathrm{relax}}$, in the polarization and relaxation processes, as well as the respective capacitance values calculated as *C* = *R*_expon_/*τ*. The values of $R_{\mathrm{expon}}{}^{\mathrm{polar}}$ and $R_{\mathrm{expon}}{}^{\mathrm{relax}}$ varied from 72 to 230 kΩ. The characteristic time values varied from 1 to 8 s, and the capacitance values varied from 11 to 97 μF. The data are presented in Table S1.

#### 3.2.2. Impedance Measurements

**Figure 4:**
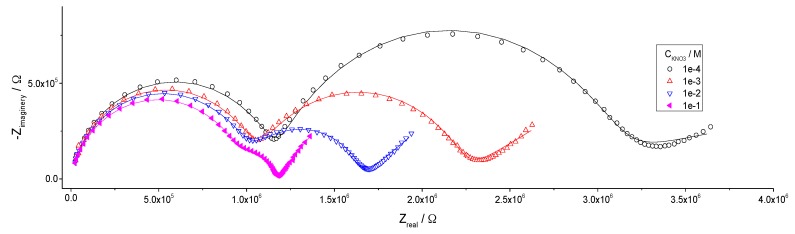
Impedance spectra of ISE 2 in contact with KNO_3_ solutions with concentrations from 10^−4^ to 0.1 M. Symbols refer to experimental data, solid lines to fitted spectra.

Like in the case of in chronopotentiometric measurements, obtaining reproducible data in the impedance measurements when one solution is replaced with another one also required several days of soaking (see Figure S2). A high-frequency semicircle in the frequency range from 100 kHz to 500–1000 Hz, followed by a low-frequency semicircle in the frequency range down to 1 Hz were present in the Nyquist plots for all ISEs studied. At even lower frequencies, a quasi-Warburg wave was present. We call this part of the spectra quasi-Warburg because it was almost linear but with slopes increasing from 0.4 for 10^−4^ M to 1.1 for 0.1 M KNO_3_. An example of the impedance spectra (data referring to ISE 2) is shown in Figure 4.

**Scheme 1:**
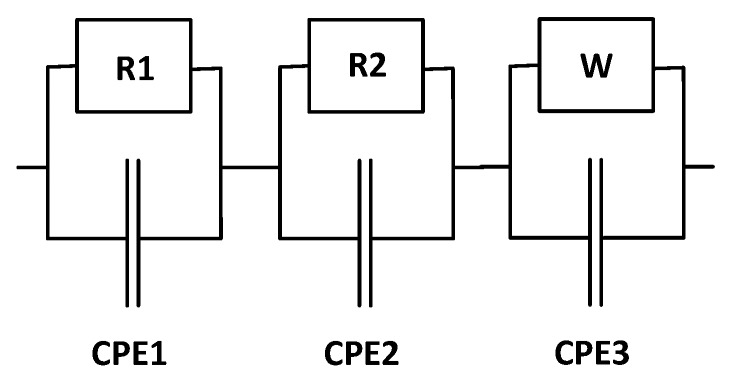
Circuit used to fit the impedance data. The high-frequency resistance (R1) and constant phase element 1 (CPE1) are registered in the frequency range from 100 kHz to 500–1000 Hz. R2 and CPE2 are the same for the frequency range from 500 to 1 Hz. The quasi-Warburg resistance (W) and CPE3 are observed at frequencies below 1 Hz.

**Figure 5:**
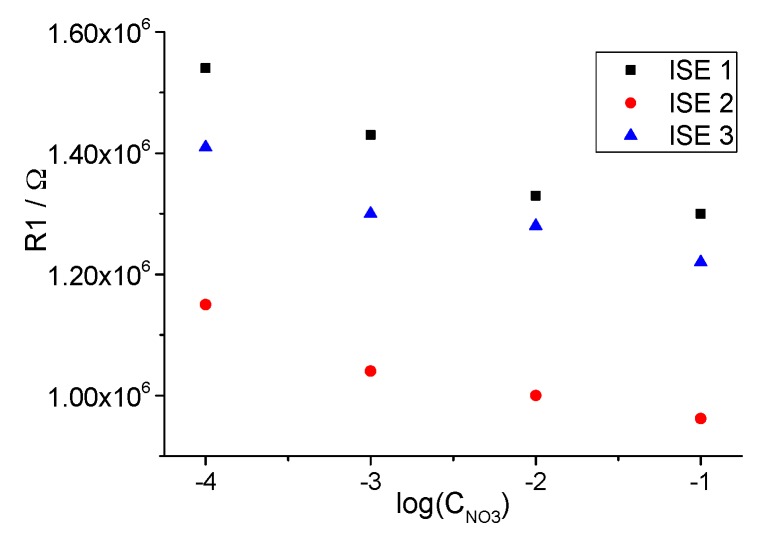
The dependence of the R1 value on the KNO_3_ concentration in the solution.

The spectra were fitted to an equivalent circuit shown in Scheme 1. Use of a constant phase element in parallel with Warburg resistance allowed for the fitting of quasi-Warburg waves. The values of R1 (the high-frequency resistance) varied from 1.6 to 1.0 MΩ, and unlike in the case of Ca^2+^-ISEs (see [21]), were not equal to the resistance values calculated from the Ohmic drops in chronopotentiometry. The value of CPE1 (constant phase element) parallel to R1 was $\left(5.4\pm 1.0\right)\times 10^{-11}\;F\times s^{n-1}$, with an *n* factor ranging from 0.89 to 0.93. The respective time constant was $\left(6.5\pm 0.7\right)\times 10^{-5}\;s$. This allowed for ascribing R1 and CPE1 to the bulk resistance and geometric capacitance of the membrane. As shown in Figure 5, the value of the bulk resistance of NO_3_^−^ selective membranes, like that of Ca^2+^-membranes [21], is not constant, and decreases over the change of the concentration of KNO_3_ from 10^−4^ to 0.1 M.

**Figure 6:**
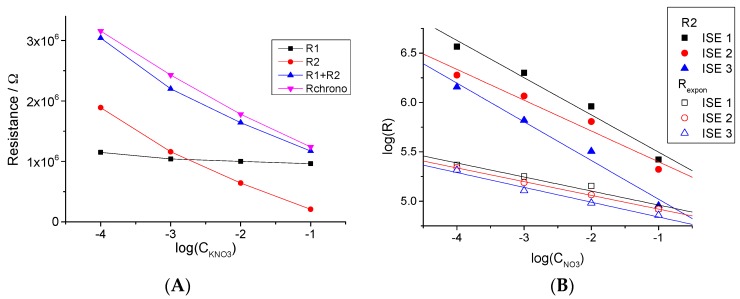
(**A**) Resistances R1, R2, R_chrono_, and the sum of R1 + R2 obtained with ISE 2; (**B**) dependence of R2 and R_expon_ on the KNO_3_ concentration in the solution for all ISEs under study.

The low-frequency resistance R2 regularly decreased over the increase of the concentration of the solution from 1–2 MΩ, when in contact with 10^−4^ M KNO_3_, to 0.1–0.2 MΩ for 0.1 M KNO_3_ (see Figure 6A). The values of CPE2 were $\left(7.9\pm 3.0\right)\times 10^{-9}\;F\times s^{n-1}$ with an *n* factor from 0.74 to 0.94. The respective time constant varied from 0.8 to 10 ms. The values of the resistance and constant phase elements are presented in Table S1. In a log/log diagram (see Figure 6B), the low-frequency resistance R2 and also the exponential resistance R_expon_ obtained by fitting the chronopotentiometric data showed linear dependence on the concentration of KNO_3_.

Altogether, the chronopotentiometric and impedance studies took two months. After that, the potentiometric measurements have been repeated, and the ISEs showed roughly the same calibration as in the beginning of the study (compare Figure 1a and Figure S3). The E^0^-s (intercepts at $\log(a_{NO3})=0$) differed from the respective initial values within ±3 mV, and the slopes were −54.9 ± 0.4 mV/ $\log(a_{NO3})$ (see Figure S4).

## 4. Discussion

The results obtained with NO_3_^−^-ISEs are similar to those obtained with Ca^2+^-ISEs [21], but not exactly the same.

The similarity refers to the dependence of the membrane bulk resistance on the concentration of the solution. Generally speaking, a decrease of the membrane bulk resistance over increased concentration of the solution may be due to co-extraction of the electrolyte. However, significant co-extraction results in deviations from the linearity of the potentiometric response, whereas the ISEs showed good linearity over the whole concentration range (10^−4^–10^−1^ M) studied by chronopotentiometric and impedance methods. Furthermore, the effect of co-extraction must be more pronounced in higher concentrations than in lower, whereas the biggest change of the bulk resistance can be seen when 10^−4^ M KNO_3_ is replaced with 10^−3^ M. In higher concentrations of KNO_3_, the change of the resistance saturates, like in the case of Ca^2+^-ISEs described earlier [21]. Thus, the origin of the non-constancy of the membrane bulk resistance within a linear response range is unclear.

On the other hand, the value of R_chrono_—the resistance obtained from the Ohmic drop in the chronopotentiometric measurements—is not consistent with R1 (the high-frequency impedance). In this respect, the properties of NO_3_^−^ ISEs differ from those of Ca^2+^-ISEs. The origin of this apparent inconsistency lies in the protocol used for the chronopotentiometric measurements. The time constants of the high- and low-frequency components in the circuit shown in Scheme 1 were about 0.07 ms and 0.8 to 10 ms, respectively. Thus, both high- and low-frequency processes are already over at 0.2 s, and the Ohmic drop in chronopotentiometric experiments refers to the sum of R1 and R2. Data shown in Figure 6A confirm this explanation.

Linear dependence (log/log) of R2 on C_KNO3_ with slopes of −0.31 to −0.39 (see Figure 6B) suggests that R2 refers to the kinetics of NO_3_^−^ ion transfer across the membrane/solution interface. The values of CPE2 confirm this interpretation; it was shown that for plasticized PVC membranes in aqueous solutions, one should expect double layer capacitance of the order of 10^−8^ F/cm^2^ [26].

Interestingly, the values of R_expon_ obtained by fitting of the polarization and relaxation parts of the chronopotentiometric curves are also regularly dependent on the KNO_3_ concentration (see Figure 6B). However, the slopes are rather low, about −0.15, and the respective capacitance are rather high, at 10 to 90 μF. The nature of the exponentially decaying process registered in chronopotentiometric measurements requires further studies.

The second term in Equation (4) is linearly dependent on the square root of time. This suggests that this term originates from diffusional limitations, and it was shown elsewhere that these limitations refer to the membrane phase [27]. One can assume that the mobility of NO_3_^−^ and TDA^+^ in the membrane phase is roughly the same. The resistivity *ρ* of the membrane, as well as the diffusional polarization, depends on *C_ion_* (the concentration of dissociated ions) and on *D_ion_* (their diffusion coefficients) as $\rho \sim {\left(C_{ion}D_{ion}\right)}^{-1}$ and $d\eta /dt^{1/2}\sim C_{ion}{}^{-1}D_{ion}{}^{-1/2}$. This allows for the estimation of *C_ion_* and *D_ion_* separately:

(5) $$C_{ion}=\frac{32RT}{\pi F^{2}}\rho i^{2}{\left(d\eta /dt\right)}^{-2}$$

(6) $$D_{ion}=\frac{\pi }{64}\rho^{-2}i^{-2}{\left(d\eta /dt\right)}^{2}$$

The estimated values are $C_{ion}=\left(3.1\pm 1.7\right)\cdot 10^{-4}$ M and $D_{ion}=\left(5.7\pm 2.3\right)\cdot 10^{-8}$ cm^2^/s. The estimate of *C_ion_*, according to the Gouy–Chapman theory, corresponds to the double layer capacitance value of about $C_{dl}=4.5\times 10^{-8}$ F/cm^2^. This value is fairly consistent with CPE2 (note: CPE2 = *C_dl_*/2 due to the symmetry of the cell). The value of *C* also gives an estimate of the association constant of TDANO_3_ (logK≃5.7), which is close to our estimate for TDABr (logK≃6.5) obtained by the segmented sandwich method [28]. The estimate for *D_ion_* is consistent with values obtained for plasticized PVC membranes by radiotracer studies [29].

## 5. Conclusions

The main goal of this study was to check whether the bulk resistance of anion-selective electrodes (using NO_3_^−^-ISEs as model system) is dependent or independent of the solution concentration within the Nernstian response range. According to our results, the ISE bulk resistance does depend on the solution concentration. This is in line with our results obtained with K^+^ and Ca^2+^-ISEs [20,21], and not in line with the current views on the mechanism of the ISE response, and in particular, on the role of the ion-exchange process at the membrane/solution interface. The origin of the dependence of the bulk resistance on the concentration of the solution requires further study.

Nitrate ISEs showed relatively high interfacial charge transfer resistance, comparable to that registered for Li^+^-ISEs [26]. Because of this, the Ohmic drop in chronopotentiometric measurements with time resolution of 0.2 s referred to the sum of the bulk and the interfacial resistances. This complication was resolved by the comparison of the results of chronopotentiometric and impedance measurements.

Combining the data on the membrane bulk resistance and the diffusional component of the polarization and relaxation curves in chronopotentiometric measurements allowed for the estimation of the concentration of ionized species (NO_3_^−^ and TDA^+^) and, respectively, for the TDANO_3_ association constant, as well as for the species diffusion coefficients in the membrane.

## Supplementary Materials

The following are available online at http://www.mdpi.com/1424-8220/18/7/2062/s1, Figure S1: The electrode design; Figure S2: Changes of the impedance spectrum of ISE 2 in contact with 0.001 M KNO_3_ solution during soaking; Figure S3: ISEs response curves over the sequential 10-fold dilution of 0.1 M KNO_3_ with DI water. The curves obtained with ISEs aged two months, when all chronopotentiometric and impedance studies were over; Figure S4: ISEs calibration curves over the sequential 10-fold dilution of 0.1 M KNO_3_ with DI water. The curves obtained with ISEs aged two months, when all chronopotentiometric and impedance studies were over. Straight line refers to ISE 1; Table S1: Electrochemical properties of the ISEs under study.
