# Supplementary material for: Electrochemical Properties of Nitrate-Selective Electrodes: The Dependence of Resistance on the Solution Concentration
Source: Sensors (Basel). 2018 Jun 28;18(7):2062. doi: 10.3390/s18072062 (PMC6069089; doi:10.3390/s18072062)
Supplement: Supplementary file 1 [file sensors-18-02062-s001.zip › sensors-316705-supplementary.docx]

**Supplementary Materials**

Electrochemical properties of nitrate-selective electrodes: the dependence of the resistance on the solution concentration

Arina Ivanova and Konstantin Mikhelson

**Figure S1**. The electrode design.

**FigureS2**. Changes of the impedance spectrum of ISE 2 in contact with 0.001 M KNO_3_ solution during soaking.

**Figure S3**. ISEs response curves over the sequential 10-fold dilution of 0.1 M KNO_3_ with DI water. The curves obtained with ISEs aged 2 months, when all chronopotentiometric and impedance studies were over.

**Figure S4**. ISEs calibration curves over the sequential 10-fold dilution of 0.1 M KNO_3_ with DI water. The curves obta**i**ned with ISEs aged 2 months, when all chronopotentiometric and impedance studies were over. Straight line refers to ISE 1.

**Table S1**. Electrochemical properties of the ISEs under study.

| **ISE** | **C_KNO3_ (М)** | **R_chrono_, MΩ** | **R_1_, MΩ** | **CPE_1_∙10^11^ F∙s^1-n1^** | **n1** | **R_2_, MΩ** | **CPE_2_∙10^9^ F∙s^1-n1^** | **n2** | **R_expon_, MΩ** | **C_expon_, μF** | **C_ion_∙10^4^, М** | **D_ion_∙10^8^, cm^2^/s** |
| --- | --- | --- | --- | --- | --- | --- | --- | --- | --- | --- | --- | --- |
| 1 | 10^‒4^ | 3.49 | 1.54 | 4.08 | 0.920 | 1.96 | 7.80 | 0.815 | 0.23 | 10.8 | 1.79 | 4.24 |
| 2 |  | 3.16 | 1.15 | 5.82 | 0.902 | 1.89 | 1.05 | 0.842 | 0.22 | 12.1 | 1.44 | 5.85 |
| 3 |  | 2.66 | 1.41 | 4.25 | 0.912 | 1.24 | 0.92 | 0.878 | 0.21 | 14.0 | 3.64 | 2.73 |
|  | Mean | 3.10 | 1.37 | 4.7 | 0.91 | 1.70 | 3 | 0.85 | 0.22 | 12.3 | 2.3 | 4.3 |
|  | SD | 0.30 | 0.14 | 0.7 | 0.01 | 0.30 | 3 | 0.02 | 0.01 | 1.1 | 0.9 | 1.0 |
|  |  |  |  |  |  |  |  |  |  |  |  |  |
| 1 | 10^‒3^ | 3.08 | 1.43 | 3.66 | 0.931 | 1.66 | 7.02 | 0.816 | 0.18 | 16.3 | 1.78 | 4.83 |
| 2 |  | 2.43 | 1.04 | 5.66 | 0.908 | 1.16 | 11.4 | 0.813 | 0.15 | 23.1 | 1.59 | 6.88 |
| 3 |  | 2.04 | 1.30 | 4.54 | 0.918 | 0.66 | 10.8 | 0.846 | 0.13 | 27.5 | 4.04 | 3.22 |
|  | Mean | 2.5 | 1.26 | 4.6 | 0.92 | 1.2 | 9.7 | 0.83 | 0.15 | 22 | 2.5 | 5.0 |
|  | SD | 0.4 | 0.14 | 0.7 | 0.01 | 0.3 | 1.8 | 0.01 | 0.02 | 4 | 1.0 | 1.3 |
|  |  |  |  |  |  |  |  |  |  |  |  |  |
| 1 | 10^‒2^ | 2.33 | 1.33 | 4.48 | 0.908 | 0.91 | 11.2 | 0.739 | 0.14 | 29.4 | 1.91 | 5.93 |
| 2 |  | 1.78 | 1.00 | 6.69 | 0.902 | 0.64 | 11.3 | 0.810 | 0.12 | 42.1 | 2.18 | 6.86 |
| 3 |  | 1.65 | 1.28 | 5.20 | 0.900 | 0.32 | 11.2 | 0.857 | 0.10 | 44.7 | 8.45 | 1.90 |
|  | Mean | 1.9 | 1.20 | 5.5 | 0.900 | 0.62 | 11.23 | 0.80 | 0.12 | 39 | 4 | 5 |
|  | SD | 0.3 | 0.14 | 0.8 | 0.003 | 0.20 | 0.04 | 0.04 | 0.01 | 6 | 3 | 2 |
|  |  |  |  |  |  |  |  |  |  |  |  |  |
| 1 | 10^‒1^ | 1.62 | 1.30 | 6.90 | 0.888 | 0.26 | 4.67 | 0.900 | 0.084 | 96.9 | 2.22 | 7.35 |
| 2 |  | 1.24 | 0.96 | 7.69 | 0.890 | 0.21 | 8.56 | 0.872 | 0.083 | 95.5 | 1.41 | 1.52 |
| 3 |  | 1.35 | 1.22 | 5.91 | 0.893 | 0.09 | 9.07 | 0.947 | 0.072 | 82.9 | 6.22 | 3.15 |
|  | Mean | 1.40 | 1.16 | 6.8 | 0.890 | 0.19 | 7.4 | 0.91 | 0.08 | 92 | 3 | 4 |
|  | SD | 0.14 | 0.13 | 0.6 | 0.002 | 0.06 | 1.8 | 0.03 | 0.01 | 6 | 2 | 2 |
